# Protocol for a scoping review of work system design in health care

**DOI:** 10.12688/f1000research.128913.1

**Published:** 2023-01-09

**Authors:** Oladunni Sarah Okunade, Victor O. Oladokun, Chinwe Juliana Iwu-Jaja, Anelisa Jaca, Charles Shey Wiysonge

**Affiliations:** 1 Cochrane South Africa, South African Medical Research Council, Parow Valley 7500, Cape Town, South Africa; 2 Department of Industrial and Production Engineering, Faculty of Technology, University of Ibadan, Oduduwa Road, Ibadan, 200132, Nigeria; 3 HIV and other Infectious Diseases Research Unit, South African Medical Research Council, Overport 4091, Durban, South Africa; 4 Division of Epidemiology and Biostatistics, Faculty of Medicine and Health Sciences, Stellenbosch University, Tygerberg 7505, Cape Town, South Africa; 5 School of Public Health and Family Medicine, University of Cape Town, Observatory 7925, Cape Town, South Africa

**Keywords:** Health care, Work system design, Work design, Work place design, Macroergonomics; Time and motion study, System engineering, Human factors

## Abstract

Background: Delivery of safe and reliable healthcare to patients and the healthcare workforce shortage amidst growing demand has been major challenge to the healthcare system. Addressing this challenge calls for designing or redesigning of healthcare work system. Work system design which is usually associated with productivity in manufacturing offers a wide spectrum of applicability in addressing this challenge of healthcare system. Despite the availability of primary studies on work system design in healthcare, there are sparse published reviews in specific contexts. This scoping review explores the existing evidence to understand the state of the art of work system design in healthcare.

Methods: The scoping review adopts the methodology of Joanna Briggs Institute for scoping review which is based on the methodological framework of Arksey and O’Malley. The search will be done on PubMed, Scopus, and Web of Science for the identification of eligible studies. A grey literature search will also be performed. A two-phase screening and extraction of data will be done by two independent reviewers. Data extraction will be done on a pre-piloted data extraction form. The findings will be presented in tables, figures, and a narrative summary. The scoping review will highlight the state of the art, gaps in knowledge and provide directions for future research.

Ethics and dissemination: This is a scoping review of primary studies and therefore ethical approval is not required. The report of the findings will be presented in line with the PRISMA reporting guidelines for scoping reviews (PRISMA-ScR). The results will be submitted to a peer-reviewed scientific journal for publication and presented at relevant conferences.

## Introduction

The provision of safe and reliable healthcare for patients with an adequate and accessible health workforce is the bedrock of an efficient and effective healthcare work system. The ability of the healthcare system to deliver safe and reliable healthcare to patients is vital to earning public trust. ^1^ Delivery of safe and reliable healthcare to the patient has been a challenge due to ineffectiveness and inefficiencies in the performance of the healthcare work system thereby resulting in patient safety problems.

Adequate and accessible health workforce is fundamental to an integrated and effective health system and provision of care, however, there is a global projection of a shortfall of 15 million health workers by 2030, with a major proportion in the low and middle-income countries. ^1^ Poor design of the healthcare work environment also contributes to the shortage of health workers. The shortage of nurses has been linked to the inability to attract and retain nurses because of poor working condition. ^2^^,^^3^ Carayon *et al*. (2012) ^4^ argue that the working conditions of healthcare professionals and workers are sources of stress, burn out and dissatisfaction. Poor infrastructure, unsafe environment, and unfair distribution of incentives among other factors are accountable for poor working conditions of health care personnel. ^5^

Addressing these challenges of the inability of the healthcare system to deliver safe and reliable health care to patients and workforce shortage effectively amidst growing demand for healthcare services calls for designing or redesigning of healthcare work system. Work system design which is usually associated with productivity improvement in the manufacturing sector offers a wide spectrum of applicability in addressing the challenges of healthcare systems. ^6^^–^^12^ The knowledge and application of work system design characteristics and principles provide the basis for healthcare organizations to engage in work improvements that can ultimately result in a variety of positive outcomes such as reduced turnover for organizations, increased job satisfaction for the workers and improved quality and safety of care for patients and their caregivers. ^4^^,^^13^^–^^16^ The term “system” as used in healthcare literature refers to an entity that aids the improvement of quality of care. ^17^^–^^20^ But the concept of “system” from Industrial and Systems Engineering point of view, also positioned by Carayon *et al*. ^4^ refers to “various elements of work that a healthcare person uses, encounters or experiences in order to perform his/her job”. The system approach considers all elements of the system and their interconnections as well as the layout of the system in ensuring the goal(s) of the system is achieved. ^21^

Work system design (WSD) involves a systematic approach that considers various components that make up the work system. It aims at minimizing or eliminating the negative aspects of work which contribute to the poor performance of the work system. ^4^ Work system design ensures that the work environment is designed for workers to have optimal workload, human safety, health and well-being ensured and the overall system performance is optimised. ^22^ The design of work systems has benefited greatly from the principles, theories, tools and techniques of Human Factors, Systems and Method Engineering. Human Factors Engineering has been identified by the National Academy of Engineering and the Institute of Medicine as an important tool for designing better healthcare systems. ^4^^,^^23^ This suggests work system design is a robust tool that has potential in improving the healthcare system. However, there is a need to investigate work system design approaches that have been used in health care.

Despite the availability of primary research studies on work system design in healthcare, there are sparse published reviews. Also, published reviews considered specific context in the application of work system design to healthcare. For instance, reviews have explored the use of human factors and ergonomics approach to work system design in healthcare. ^24^^,^^25^ Also, a review has demonstrated the use of the Systems Engineering Initiative for Patient Safety (SEIPS) model to improve patient work. ^26^ To the best of our knowledge, a review with focus on work system design in healthcare in a broader context has not been conducted. A comprehensive understanding of work system design in healthcare is vital to exploring its approaches to addressing the challenges of the healthcare system. Consistent with scoping review which is known for mapping out the body of literature on a topic area, ^27^ this scoping review aims to explore the existing evidence to understand the state of the art of work system design in healthcare. To achieve this aim, the review objectives are:

1. to report work system design approaches in healthcare
2. to identify areas where it has been applied in healthcare
3. identify gaps for further research and make recommendations for future research.

## Methods

### Protocol design

The scoping review adopts Joanna Briggs Institute (JBI) scoping review methodology which is based on the methodological framework of Arksey and O’Malley. ^28^^,^^29^ The methodology has 6 stages which are as follows:

Stage 1. identification of the research question;

Stage 2. identification of relevant studies;

Stage 3. selection of eligible studies;

Stage 4. charting the data;

Stage 5. collating, summarising and reporting the results;

Stage 6. consultation with stakeholders,

Stage 6 of the methodology, although optional, is valuable if it can be explored. This scoping review will not consider it because of constraints of budget and time. Also, the Preferred Reporting Items for Systematic reviews and Meta-Analyses extension for Scoping reviews (PRISMA-ScR) checklist was followed in preparing the protocol.

### Identifying the research questions

The research questions were developed using the iterative process of Arksey and O’Malley through consultation with the team and the team came up with the following questions: (1) What are the work system design approaches used in healthcare? (2) Which areas have work system designs been applied in healthcare? (3) What are the gaps and recommendations for future research?

### Identification of relevant studies

Population, Concept, and Context (PPC) framework was used to determine relevant terms and studies. ^28^ The search will identify all studies without any restriction to date and geographical location. The search will be implemented across three electronic databases: PubMed, Scopus, and Web of Science. These databases were selected for comprehensiveness and coverage of a broad range of disciplines. The search strategy consists of keywords generated from related studies and are approved by all authors to describe the scoping review and its methodology: work system design, work system, system engineering, human factors, work design, workplace design, job design, organisation design, macroergonomics, work organisation, time and motion study, healthcare, health system, health care. The preliminary search on PubMed (see Table 1) combines keywords with Boolean operators. This will be adapted for search in other databases in consultation with a librarian.

**Table 1.** Search strategy for PubMed.

Search #	Search strategy
#3	#1 AND #2
#2	TITLE-ABS (healthcare OR “health care” OR “health system” OR “healthcare systems”)
#1	TITLE-ABS (“work system design” OR “work system” OR “system engineering” OR “work design” OR “workplace design” OR “job design” OR “organizational design” OR macroergonomics OR work AND organization AND time OR “motion study”)

The same search strategy will be used for a web search to be conducted on Google Scholar to identify grey literature. In consideration of screening time for each hit and the likelihood of not yielding many more relevant articles, the decision to screen only the first 50 hits was made. Other identified websites such as National Academy of Engineering, Ergonomics and Human factors organization will be manually searched. The studies to be included in this scoping review must meet the following inclusion and exclusion criteria as depicted in Table 2.

**Table 2.** Inclusion and exclusion criteria.

	Inclusion criteria	Exclusion criteria
Focus	Primary studies on Work system design in healthcare	Exclude all review articles
Context	Global	
Language	English	Other languages
Publication year	No restriction on the study publication year	

The Population/Concept/Context (PCC) framework is outlined to guide in the identification and screening of relevant studies. The population which identifies important characteristics of participants is not applicable to this study. The concept is focused on exploring the existing evidence in understanding work system design approaches as applied to healthcare. Primary studies on work system design in healthcare will be included. All reviews whether literature or systematic will be excluded. The context for the scoping review is global and studies in the English language only will be included while all studies in other languages will be excluded. There is no date limit to this study.

### Selection of eligible studies

The screening process is in two phases; screening of titles and abstracts and screening of full texts. Two independent reviewers will carry out screening of titles and abstracts, to select studies that are in line with PPC framework as given in the inclusion and exclusion criteria. Full-text screening is the second phase to be done by two independent reviewers to select studies that met the inclusion criteria for this scoping review. At this phase, full-text studies will be excluded if they do not meet inclusion requirements and the reason for excluding the studies will be provided in the final report. Data generated by two independent reviewers will be extracted, assessed and discussed. The report of the final results of the search will be presented following the Preferred Reporting Items for Systematic Reviews and Meta-Analysis (PRISMA) flow diagram. Disagreements in the selection process by the two independent reviews will be resolved through dialogue or a third-party reviewer. Consensus will be used in resolving disagreement but if it is not achieved, a third reviewer will be consulted.

### Data extraction

Data extraction gives a descriptive summary of study results to address scoping review objectives and research questions. Excel spreadsheet was used in the development of a template for data extraction. The validity of the template will be piloted and tested prior to the commencement of data charting. Key information to be extracted includes authors, year, title, journal, study objectives, study setting, and study site, to mention a few. Table 3 gives the data charting framework for this scoping review. A data extraction form will be used for the collection of general information from relevant studies and data relevant to answering the research questions. Data extraction will be done by independent reviewers and consensus will be reached by team members on resolving discrepancies. Revision and update will be done on the data extraction form during the review process to accommodate necessary changes.

**Table 3.** Data charting framework.

Category	Description
Author last name	
Year and doi	
Title	
Journal	
Study objectives	This describes the stated objective of the study
Study settings	Describes the environment the study was conducted that is the area of healthcare where WSD was applied
Study Site	This gives the country where the study was conducted
Research design type	This shows the type of research design used in the study. The review will classify the types into qualitative, quantitative and a combination of qualitative and quantitative
WSD focus	This gives the component(s) of the work system that the study focused on be it the person, task, tools and technology, organisation and environment
WSD approach	This states the WSD method applied to the study and the tool used
Conclusion of the study	This describes the outcome(s) or finding(s) of the study
Limitation of Study	This describes the shortcoming(s) of the study which could be due to research design, and methodology to mention a few.

### Data synthesis strategy

The collation of findings from the data extraction form will be analysed using themes relating to the review objectives. Descriptive analysis to summarize the characteristics of the included studies such as publication year, study setting and site and also the evidence on the approaches of work system design in healthcare, the area of its applications in healthcare and the limitations of the approaches and application of work system design in healthcare. On the other hand, areas that are not well-researched and may require further investigation will also be presented. Results will be presented in form of tables and figures where appropriate. Also, a narrative summary of the findings will be presented.

This study will explore the existing evidence to understand the state of the art of work system design in healthcare by providing breadth and depth of work system design knowledge as an important tool for designing better healthcare systems. It will also identify gaps in knowledge and provide directions for future research.

### Study status

Currently developing and piloting data extraction form, and the next step will be running of the search and screening at a later date.

### Dissemination

The report of the findings will be presented in line with the PRISMA reporting guidelines for scoping reviews (PRISMA-ScR). The results will be submitted to a peer-reviewed scientific journal for publication and presented at relevant conferences and events.

## Data Availability

No data are associated with this article. Figshare: PRISMA-ScR-Fillable-Checklist-ProtocolSubmission.docx. https://doi.org/10.6084/m9.figshare.21618258.v1. ^30^
- PRISMA-ScR-Fillable-Checklist-Protocol Submission.docx Data are available under the terms of the Creative Commons Zero “No rights reserved” data waiver (CC0 1.0 Public domain dedication).
