# Supplementary material for: Protocol for a scoping review of work system design in health care
Source: F1000Res. 2023 Oct 30;12:21. Originally published 2023 Jan 9. [Version 2] doi: 10.12688/f1000research.128913.2 (PMC10966335; doi:10.12688/f1000research.128913.2)
Supplement: Peer review history

# Reviewer report: Reviewer response for version 2

**DOI:** 10.5256/f1000research.158042.r253279

**Published:** 2024-03-26

**Authors:** Irwan Budiman

**Affiliations:** 1 Universitas Prima Indonesia, Medan, North Sumatra, Indonesia

The objectives to be achieved have been well described and the protocol developed can achieve the expected goals. But, please re-check the keywords for the more suitable to this research.

 Macroergonomics is closely related to the discussion of ergonomics which focuses on the structure and organization of work direction related to tasks, content, and time factors. Structural reporting flow can be done from the operational level to the functional level, or from the functional level to the strategic / business level in health care. It would be better if the described work system improvements in health care were organized systematically up to the reporting flow. The method has also been compiled quite well. However, it would be better if the method can be described in more detail so that it can be easily replicated by others.

 Datasets have been presented in full.

Is the study design appropriate for the research question?

Partly

Is the rationale for, and objectives of, the study clearly described?

Yes

Are sufficient details of the methods provided to allow replication by others?

Yes

Are the datasets clearly presented in a useable and accessible format?

Yes

Reviewer Expertise:

Macroergonomics and Ergonomics Work System Design

I confirm that I have read this submission and believe that I have an appropriate level of expertise to confirm that it is of an acceptable scientific standard, however I have significant reservations, as outlined above.

# Reviewer report: Reviewer response for version 1

**DOI:** 10.5256/f1000research.141552.r185072

**Published:** 2023-08-01

**Authors:** Carlo Fabricatore

**Affiliations:** 1 E.H.E. Europa Hochschule EurAka CH, Therwil, Switzerland

Overall, the core structure and contents of this protocol seem sound.

 The article requires some further proofreading to improve readability and amend some sparse typos and/or improve the editing. I believe that there is need and scope to improve the protocol by considering the following points:

- The introduction proposes a valid emphasis on “work system design”. However, I would recommend strengthening the background, by introducing an overview of the work system theory, in order to better contextualize the reader by explaining what a work system is, what the involved elements and relationships are, and why their perspective is important in healthcare practice.
- By focussing on “application areas” RQ2 seems to me overly vague, and it can lead to difficulties in analyzing data from the review. It seems to me that this scoping review warrants a systematic examinations of applications of work system design (WSD) in healthcare, and that “application areas” can be one of the multiple analyses to be done based on the “application data” extracted from the reviewed studies. Other analyses should then ensue, such as, for example, how many studies have empirically corroborated the effects of WSD, types of effects ascertained, etc. A deeper and better structured analysis along these lines would then facilitate the identification of trends and gaps in current research, and the subsequent proposal of orientation for future research.
- I would recommend reporting (and further detailing) the systematic literature search protocol based on the PRISMA-S statement (Rethlefsen *et al*., 2021 ^1^), in order to improve the replicability of the search.
- The authors indicate that data extraction will be based on a pre-developed template, and that “The validity of the template will be piloted and tested prior to the commencement of data charting”. This is somewhat confusing to me:
  (a) Firstly, what do the authors mean by “charting”? If this is about identifying and classifying excerpts in the original texts based on predefined categories, then this would sound to me like a data coding procedure appropriate to extract data prior to analysis (e.g., identifying all the excerpts describing which elements of a work system have been re-designed, to later on propose a synthesis highlighting which elements have been more frequently addressed, by how many studies, etc.) If “charting” means something else to the authors, then it should be clarified and justified.
  (b) Secondly, the authors should clarify how they intend to “pilot and test” the template prior to the commencement of the data extraction process. I would also consider that data extraction templates do not need to be “fixed” a-priori. They may be modified through iterative processes of data extraction and coding, e.g., as suggested by the template analysis technique (King, 2012 ^2^).
- Consider discussing the broader relevance of the protocol: does it represent a contribution in and of itself? Can it be useful to guide other reviews?

Is the study design appropriate for the research question?

Yes

Is the rationale for, and objectives of, the study clearly described?

Partly

Are sufficient details of the methods provided to allow replication by others?

Partly

Are the datasets clearly presented in a useable and accessible format?

Not applicable

Reviewer Expertise:

Human factors engineering in healthcare and education.

I confirm that I have read this submission and believe that I have an appropriate level of expertise to confirm that it is of an acceptable scientific standard, however I have significant reservations, as outlined above.

## References

[1] : PRISMA-S: an extension to the PRISMA Statement for Reporting Literature Searches in Systematic Reviews. *Syst Rev* .2021;10(1) : 10.1186/s13643-020-01542-z 39 10.1186/s13643-020-01542-z 33499930 PMC7839230

[2] : Doing Template Analysis.2012; 10.4135/9781526435620.n24 426-450 10.4135/9781526435620.n24

# Response

**Published:** 2023-10-24

**Authors:** Oladunni Okunade

**Affiliations:** University of Ibadan, Ibadan, Nigeria

Thank you for your comments. The background of this study has been strengthened as suggested. RQ2 has been reviewed.

 The PRISMA-S as given by Rethlefsen *et al*. (2021) may not be applicable to the study because it is meant for reporting systematic review. PRISMA-ScR as stated in the protocol is appropriate PRISMA reporting guidelines for scoping review.

 The charting refers to data extraction, one of the six stages of the scoping review methodology.

 “pilot and test” template prior to the commencement of data extraction process is to ensure the validity of the process. This will be carried out on a sample of included studies in order to ensure consistency in the use of the framework for data extraction and if need be, the data extraction framework can be modified

# Reviewer report: Reviewer response for version 1

**DOI:** 10.5256/f1000research.141552.r179129

**Published:** 2023-07-04

**Authors:** Cecilia Berlin

**Affiliations:** 1 Div. of Design & Human Factors, Chalmers University of Technology, Gothenburg, Sweden

This protocol for a scoping review aims to broadly map out the scope of available work (system) design literature that is applicable to healthcare settings. Its intention, aim, proposed methodology and intended further steps are detailed and described in a sensible and mostly satisfactory way. I think the forthcoming paper sounds promising and I would be interested to read it. 

 The protocol (and its subsequent paper) will benefit from a thorough language check. The way this is written, I have a feeling that the authors are competent writers, but there are some incorrect singular/plural noun choices, and sentence structure errors here and there that could be edited for clarity, mostly by reducing length and complexity. One example is the first sentence of the 3rd paragraph ("Addressing these..."). 

 Research question 2 should grammatically be written, "Which areas have work system designs been applied _to_ in healthcare?". I would however suggest that "areas" is rather vague in this sense, so I would like to suggest "How have work system design interventions been applied in healthcare?". In this way, the focus can be placed on reporting intervention studies and empirical findings in RQ2. However, this is just a suggestion to increase clarity. 

 In your search terms, remember that there may be some variation in whether it is called "system engineering" or "systems engineering". I might also recommend searching "sociotechnical systems" and "socio-technical systems".

Is the study design appropriate for the research question?

Yes

Is the rationale for, and objectives of, the study clearly described?

Yes

Are sufficient details of the methods provided to allow replication by others?

Yes

Are the datasets clearly presented in a useable and accessible format?

Not applicable

Reviewer Expertise:

Production ergonomics, social sustainability, work system design, human factors in manufacturing, macroergonomics, cognitive ergonomics

I confirm that I have read this submission and believe that I have an appropriate level of expertise to confirm that it is of an acceptable scientific standard.

# Response

**Published:** 2023-10-24

**Authors:** Oladunni Okunade

**Affiliations:** University of Ibadan, Ibadan, Nigeria

Thank you for your comments. The article has been copyedited. RQ2 has been reviewed as suggested to increase clarity **.**
